# Determining Levers of Cost-effectiveness for Screening Infants at High Risk for Peanut Sensitization Before Early Peanut Introduction

**DOI:** 10.1001/jamanetworkopen.2019.18041

**Published:** 2019-12-20

**Authors:** Matthew Greenhawt, Marcus Shaker

**Affiliations:** 1 Section of Allergy and Immunology, Food Challenge and Research Unit, Children’s Hospital Colorado, University of Colorado School of Medicine, Aurora; 2 Section of Allergy and Immunology, Dartmouth-Hitchcock Medical Center, Lebanon, New Hampshire; 3 Dartmouth Geisel School of Medicine, Hanover, New Hampshire

## Abstract

**Importance:**

Early peanut introduction reduces the risk of developing peanut allergy, especially in high-risk infants. Current US recommendations endorse screening but are not cost-effective relative to other international strategies.

**Objective:**

To identify scenarios in which current early peanut introduction guidelines would be cost-effective.

**Design, Setting, and Participants:**

This simulation/cohort economic evaluation used microsimulations and cohort analyses in a Markov model to evaluate the cost-effectiveness of early peanut introduction with and without peanut skin prick test (SPT) screening in high-risk infants during an 80-year horizon from a societal perspective. Data were analyzed from April to May 2019.

**Exposures:**

High-risk infants with early-onset eczema and/or egg allergy underwent early peanut introduction with and without peanut SPT screening (100 000 infants per treatment strategy) using a dichotomous 8-mm SPT cutoff value (stipulated in the current US guideline).

**Main Outcomes and Measures:**

Cost, quality-adjusted life-years (QALYs), net monetary benefit, peanut allergic reactions, severe allergic reactions, and deaths due to peanut allergy.

**Results:**

In the simulated cohort of 200 000 infants and using the base case during the model horizon, a no-screening approach had lower mean (SD) costs ($13 449 [$38 163] vs $15 279 [$38 995]) and higher mean (SD) gain in QALYs (29.25 [3.28] vs 29.23 [3.30]) vs screening but resulted in more allergic reactions (mean [SD], 1.07 [3.15] vs 1.01 [3.02]), severe allergic reactions (mean [SD], 0.53 [1.66] vs 0.52 [1.62]), and anaphylaxis involving cardiorespiratory compromise (mean [SD], 0.50 [1.59] vs 0.49 [1.47]) per individual. In deterministic SPT sensitivity analyses at base-case sensitivity and specificity rates, screening could be cost-effective at a high disutility rate (the negative effect of a food allergic reaction) (76-148 days of life traded) for an at-home vs in-clinic reaction in combination with high baseline peanut allergy prevalence among infants at high risk for peanut allergy and not yet exposed to peanuts. If an equivalent rate and disutility of accidental and index anaphylaxis was assumed and the 8-mm SPT cutoff had 0.85 sensitivity and 0.98 specificity, screening was cost-effective at a peanut allergy prevalence of 36%.

**Conclusions and Relevance:**

The results of this study suggest that the current screening approach to early peanut introduction could be cost-effective at a particular health utility for an in-clinic reaction, SPT sensitivity and specificity, and high baseline peanut allergy prevalence among high-risk infants. However, such conditions are unlikely to be plausible to realistically achieve. Further research is needed to define the health state utility associated with reaction location.

## Key Points

**Question:**

Although the current US policy of early peanut introduction is not cost-effective compared with universal introduction without screening, are there variables and assumptions under which this policy could be cost-effective?

**Findings:**

In this simulation/cohort economic evaluation, screening high-risk infants before peanut introduction was cost-effective at very high disutility (76-148 days of life traded) for having an in-office index reaction or with greater than 36% baseline peanut allergy prevalence and peanut skin prick test sensitivity of 0.85 and specificity of 0.98.

**Meaning:**

These results suggest that cost-effectiveness of the current US early peanut introduction policy depends on a high disutility for having an index peanut allergic reaction at home, a high ambient prevalence of peanut allergy, and very high sensitivity and specificity of the skin prick test.

## Teaser

This economic evaluation identifies scenarios in which the current US guidelines for early introduction of peanut to infants would be cost-effective.

## Introduction

Peanut allergy affects 1% to 4.5% of children, can potentially be severe, and is not readily outgrown in most individuals.^1^ Moreover, although treatments are on the horizon, a cure remains elusive, and management involves strict avoidance and anaphylaxis preparedness.^2,3^ For these reasons, peanut allergy is associated with impaired quality of life and anxiety.^4,5^ Important advances have been made regarding prevention of peanut allergy through deliberate early introduction, in particular targeting populations of children at risk for developing peanut allergy, as demonstrated in the Learning Early About Peanut Allergy (LEAP) trial in which a dramatic risk reduction was noted against developing peanut allergy at 5 years of age through early peanut introduction at 4 to 11 months of life compared with delayed introduction.^6^

The strength of these findings helped to reverse prior recommendations to avoid peanut in infants and young children until 3 years of age and resulted in the recent National Institutes for Allergy and Infectious Disease (NIAID) addendum guidelines that recommend early introduction to prevent peanut allergy.^7^ This strategy was adopted in the United Kingdom, Canada, Australia, and New Zealand, although the wording of the policy and the implementation of this guidance vary among these nations. The United Kingdom, Australia, and New Zealand, and now the Canadian Pediatric Society all recommend early peanut introduction at approximately 6 months of age (but not before 4 months of age) without any prescreening and risk stratification.^7,8,9,10^ However, the NIAID guidelines strongly recommend that high-risk infants (eg, those with severe eczema and/or egg allergy) undergo peanut allergy testing at 4 to 6 months of age before having peanut introduced. Infants demonstrating peanut skin prick test (SPT) sensitization ranging from 3 to 7 mm are recommended to have in-clinic peanut introduction; those with sensitization of at least 8 mm are diagnosed as having preexisting peanut allergy, and introduction is withheld. For lower-risk infants (or infants not at risk), peanut introduction is advised as early as 6 months of age, without such medicalization or screening, in accordance with family values and preferences^7^ (eTable in the Supplement).

Although the NIAID strategy largely follows, with some extension, the parameters used in the LEAP study, the necessity of medical screening before peanut introduction was never specifically evaluated (because screening was not a randomized study variable) and can be questioned in light of the differing international strategies chosen.^11^ Indeed, applying the NIAID criteria to the HealthNuts population (an Australian population-level food allergy prevalence study in children aged 1 year),^12^ even if all infants with early-onset eczema and/or egg allergy were screened (approximately 16% of all infants born each year), 23% of all children in this cohort diagnosed with peanut allergy would have been missed. This raises questions about the sensitivity and specificity of these criteria as well as their necessity given that most initial reactions to peanut were mild and a fatal index peanut reaction has never been described.^13^

Moreover, in a recent cost-effectiveness model exploring the differing international recommendations for how to implement early peanut introduction, the screening strategy (irrespective of use of serologic evaluation or SPT) was dominated by a no-screen approach (eg, screening resulted in higher costs and lower benefits). Screening led to greater overdiagnosis, which attenuated the benefit of preventing a peanut index reaction.^14^ This overdiagnosis is a residual result of using a skin test cutoff based on a probability for allergy (whereby some will be diagnosed without ever eating and reacting to peanut), whereas if these individuals were allowed to ingest peanut, not all of them would have a reaction per se. Cost-effectiveness of the recommendations may not affect their implementation, because caregivers and physicians may still opt for care that is considered wasteful or low value based on such findings. A limitation of the previous analysis^14^ was that it strictly compared the approaches and did not explore all levers or pathways that may exist for the NIAID recommendations to potentially be cost-effective. One particular lever of cost-effectiveness may be the health utility surrounding the location where someone’s index reaction to peanut occurs. In the case of early introduction, some families and health care professionals may differentially value or more strongly prefer a particular setting for where a first potentially severe index reaction attributable to early peanut introduction occurs—in a clinic under medical supervision vs at home.^15^ Caregivers and health care professionals who place high value on avoiding an at-home index reaction to peanut, whether mild or severe, and who would rather this reaction occur in a medically supervised setting may strongly prefer the recommended screening approach with reflexive food challenge for modest positive screening results (3- to 7-mm wheal of a peanut SPT), whereas those who value this scenario less or have no preference may opt for at-home introduction. Such differing valuation could drastically affect the cost-effectiveness of screening. Therefore, we undertook this simulation and cost-effectiveness analysis to evaluate the optimal peanut introduction strategy for high-risk infants in the setting of differential potential health utility for medically supervised vs at-home index reactions to peanut.

## Methods

This study was deemed exempt from institutional review board approval and informed consent by the Colorado Multiple Institutional Review Board of the University of Colorado because it evaluated simulated cohorts of infants at risk for peanut allergy with the use of aggregate published data as model inputs and did not qualify as human research. The analysis conformed to the Consolidated Health Economic Evaluation Reporting Standards (CHEERS) reporting guideline.^16^

### Decision Model

Microsimulations (100 000 per strategy) and cohort analyses were used to evaluate a Markov model of early peanut introduction with and without peanut SPT screening in infants deemed to be at high risk for peanut allergy development per the NIAID guidelines (those with early-onset eczema and/or egg allergy) during an extended 80-year horizon from a societal perspective. An extended time horizon was used to better understand the long-term societal outcomes of screening decisions made during infancy in an allergy considered to be lifelong for most patients.

**Figure 1.**
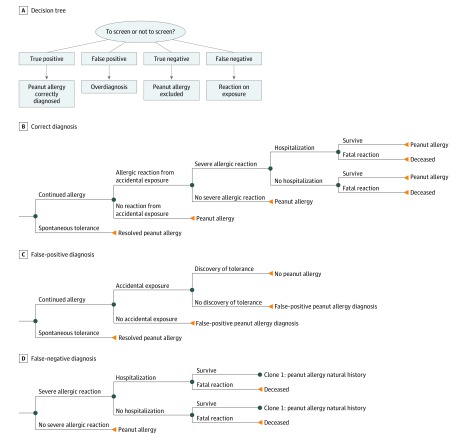
Outcomes of Peanut Allergy Screening and Decision Trees of Diagnoses

Infants randomized to screening received a peanut SPT with an initial dichotomous outcome defined at 8 mm, the cutoff in the NIAID guidelines at which an infant is recommended to be diagnosed as allergic and not offered early introduction. A positive test result was considered to be a wheal of at least 8 mm; a negative test result, a wheal of less than 8 mm. Children with an SPT result of 3 to 7 mm underwent supervised peanut challenge in an allergy clinic, and those with an SPT result of less than 3 mm underwent home peanut introduction, per NIAID guidelines.^7,14^ Figure 1 depicts the following outcomes of screening at the 8-mm threshold: (1) true-positive (sensitized and allergic), (2) false-positive (sensitized but not truly allergic; however, not challenged to determine this), (3) true-negative (not sensitized and tolerant), and (4) false-negative (skin test <8 mm but allergic). Sensitivity of peanut SPT (≥8 mm) was derived from the HealthNuts cohort and modeled at 0.54 with a specificity of 0.98, the population from which the NIAID guideline cutoff value was obtained.^17^ In the base-case model, a false-positive test result (SPT ≥8 mm) led to a diagnosis of peanut allergy without challenge (as per NIAID guidelines)^7^; however, infants with a false-positive SPT result did not assume the risks of allergic reactions from accidental peanut ingestion. Children with true-positive test results (SPT ≥8 mm) avoided peanut and entered the natural history model of peanut allergy, whereas those with false-positive results avoiding peanut entered a natural history model that included avoidance under a peanut allergy health state utility but also included the possibility that natural tolerance would be inadvertently discovered. Children with false-negative SPT results were presumed to discover (through supervised or home challenge) that they were allergic within the initial year of the model cycle and subsequently entered the peanut allergy health state. Models were evaluated for cost, quality-adjusted life-years (QALYs), net monetary benefit, peanut allergic reactions, severe reactions, and deaths due to peanut allergy. Model trackers were used to evaluate episodes of severe allergic reactions and fatalities.^12,18,19,20,21^ A threshold for cost-effective care was set at $100 000/QALY.^22^

### Probabilities

Model inputs^12,13,17,19,20,21,23,24,25,26,27,28,29,30^ shown in Table 1 included a 14% prevalence of peanut allergy in the high-risk infants during infancy.^13,14^ Age-adjusted all-cause mortality was incorporated with 2013 US life tables.^23^ The accidental rate of peanut exposure was 11.7% per year (range, 5.0%-45.0%), with severe allergic reactions occurring in 52.0% of accidental reactions (range, 1.0%-55.0%). Severe reactions on first exposure to peanut occurred in 30.5% of patients (range, 5.0%-55.0%), with 8.3% of index reactions involving respiratory or cardiovascular compromise.^12,20^ Hospitalization was required in 35.0% of patients experiencing severe allergic reactions (range, 5.0%-45.0%).^21^ Deaths due to food allergy were included (aged 0-19 years, 3.25 [95% CI, 1.73-6.10] per 1 million person-years; aged ≥20 years, 1.81 [95% CI, 0.94-3.45] per 1 million person-years).^19^ A 20% rate of discovery of overdiagnosis (range, 5%-80%) was modeled during the first 20 years of the simulation.

**Table 1.** Simulation Model Inputs

Variable	Model Reference	Source
US life table	National Vital Statistics Reports, April 2017	Arias et al,^23^ 2017
Testing characteristics	SPT (8-mm cutoff): sensitivity, 0.54 (range, 0.50 to 0.98); specificity, 0.98 (range 0.60 to 0.99)	Peters et al,^17^ 2013
Deaths due to food allergy	Aged 0-19 y, 3.25 (95% CI, 1.73 to 6.10) per 1 million person-years (sensitivity, 0.30 to 30.00); aged ≥20 y, 1.81 (95% CI, 0.94 to 3.45) per 1 million person-years (sensitivity, 1.81 to 18.10)	Umasunthar et al,^19^ 2013
Rate of accidental peanut exposure and symptoms in persons with peanut allergy	11.7%/y (sensitivity, 5.0% to 45.0%)	Vander Leek et al,^20^ 2000
Rate of severe allergic reaction in persons with peanut allergy per year	Accidental: 52.0% (sensitivity, 1.0% to 55.0%); index introduction: 30.5% (sensitivity, 5.0% to 55.0%)	Vander Leek et al,^20^ 2000; Osborne et al,^12^ 2011
Hospitalization rate after ED visit for severe allergic reaction	35.0% (sensitivity, 5.0% to 45.0%)	Robinson et al,^21^ 2017
Cost of primary health care visits per year	$100 (Sensitivity, $94 to $105)	Gupta et al,^27^ 2013; US Department of Labor, Bureau of Labor Statistics,^24^ 2017
Cost of allergist visits for food allergy	Initial consultation for testing: $687 (sensitivity, $500 to $1200); oral food challenge; $124 (sensitivity, $100 to $600); annual follow-up visits: $149 (sensitivity, $140 to $152)	Gupta et al,^27^ 2013; CMS,^25^ 2017; Dartmouth-Hitchcock,^26^ 2017; US Department of Labor, Bureau of Labor Statistics,^24^ 2017
Cost of nutritionist visits for food allergy per year	$17 (Sensitivity, $15 to $18)	Gupta et al,^27^ 2013; US Department of Labor, Bureau of Labor Statistics,^24^ 2017
Cost of alternative health care professional visits for food allergy per year	$25 (Sensitivity, $22 to $27)	Gupta et al,^27^ 2013; US Department of Labor, Bureau of Labor Statistics,^24^ 2017
Incremental cost of groceries (living with food allergy) per year	$310 (Sensitivity, $290 to $330)	Gupta et al,^27^ 2013; US Department of Labor, Bureau of Labor Statistics,^24^ 2017
Costs of job-related opportunity due to food allergy per year	$2597 (Sensitivity, $0 to $2697)	Gupta et al,^27^ 2013; US Department of Labor, Bureau of Labor Statistics,^24^ 2017
Cost of personal epinephrine autoinjector	$715 (Sensitivity, $50 to $1000)	Shaker et al,^29^ 2017; US Department of Labor, Bureau of Labor Statistics,^24^ 2017
Cost of skin test	$24 (Sensitivity, $10 to $40)	CMS,^25^ 2017
Cost of hospitalization	$5899 (95% CI, $5732 to $6066)	Patel et al,^28^ 2011; US Department of Labor, Bureau of Labor Statistics,^24^ 2017
Cost of ED visit	$691 (95% CI, $689 to $693)	Patel et al,^28^ 2011; US Department of Labor, Bureau of Labor Statistics,^24^ 2017
Negative health state influence for food allergy and anaphylaxis	−0.09 (Sensitivity, −0.05 to −0.11)	Carroll and Downs,^30^ 2009
Cycle length	1 y	NA
Peanut allergy pretest probability	14.0% (Sensitivity, 14.0% to 40.0%)	Koplin et al,^13^ 2016
Annual discount rate	0.03 (Sensitivity, 0 to 0.05)	NA
Probability of identifying false-positive test result during model horizon	20.0% (Sensitivity, 5.0% to 80.0%)	NA

Abbreviations: CMS, Centers for Medicare & Medicaid Services; ED, emergency department; NA, not applicable; SPT, skin prick test.

### Costs

Costs of living with peanut allergy were expressed in 2018 dollars and discounted at 3% per annum. Job-related opportunity costs of caregivers were estimated at $2597 per year.^24,25,26^ Direct costs included allergist, primary health care professional, nutritionist, and alternative health care professional visits, self-injectable epinephrine, groceries, and anaphylaxis management (emergency department care and hospitalization), which were obtained from previously published analyses.^27,28,29,31^

### Health State Utilities

Quality-adjusted life-years were derived from health state utilities for patients living with peanut allergies, discounted at 3% per annum. Carroll and Downs^30^ assessed health state utility values by standard gamble and time trade-off in 4016 parents or guardians of at least 1 child younger than 18 years recruited at random from multiple sources. Health state utility values for moderate and severe allergic reactions were 0.93 for standard gamble and 0.91 for time trade-off. The disutility of an allergic reaction was −0.09.^30^ Health state disutility represents a negative health detriment assigned for a particular event relative to the condition of interest. In this case, the disutility translates to approximately 33 days of life in a single year being traded to avoid having an allergic reaction.

We used SDs to describe probabilistic determination of uncertainty associated with variation in event rates resulting from linked probabilities of individual outcomes during Monte Carlo simulation. In addition, for probabilistic sensitivity analysis, triangular distributions (minimal, maximal, and mode values specified) were evaluated simultaneously to evaluate certainty of findings.

### Statistical Analysis

Data were analyzed from April to May 2019. Univariate deterministic sensitivity analyses were performed on individual variables. Multivariate probabilistic sensitivity analyses (n = 1000) with triangular modal distributions were performed across upper and lower bounds of plausible ranges. Sensitivity analyses included SPT sensitivity ranges from 0.70 to 0.98 and specificities of 0.33 to 0.99, higher baseline prevalence rates of peanut allergy among high-risk infants undergoing early peanut introduction, lower chances of discovering false-positive diagnoses, and mortality rates increased to 10-fold in the base-case risk.^17^ Accidental annual peanut reaction rates were modeled to a lower limit of 1%, with as many as 55% of index reactions resulting in anaphylaxis. Sensitivity analyses excluding job-related opportunity costs and evaluating epinephrine autoinjector costs at $50 per year were also performed.

## Results

The simulated population included 100 000 infants with and 100 000 infants without the SPT screening. During the 80-year time horizon, a no-screening approach dominated SPT screening in high-risk infants for costs (mean [SD], $13 449 [$38 163] vs $15 279 [$38 995]) and QALYs (mean [SD], 29.25 [3.28] vs 29.23 [3.30]). As shown in Table 2, when compared with screening, a no-screening approach resulted in slightly higher rates of allergic reactions (mean [SD], 1.07 [3.15] vs 1.01 [3.02]), severe allergic reactions (mean [SD], 0.53 [1.66] vs 0.52 [1.62]), and accidental anaphylaxis together with index reactions that included respiratory or cardiovascular compromise (mean [SD], 0.50 [1.59] vs 0.49 [1.47]) per patient at risk. However, rates of deaths due to food allergy were similar (and rare). Skin testing led to peanut allergy diagnosis in 6.5% of the screening cohort vs 6.3% of participants in the nonscreening cohort as the model concluded. When modeling the possible protective benefit of screening against an index reaction–associated peanut fatality, a no-screening approach continued to dominate the analyses, even assuming as much as a 1000-fold protection against fatality on the index ingestion associated with screening (screening incremental cost, $1532; effectiveness, −0.018 in the cohort analysis).

**Table 2.** Comparison of Screening vs No Screening Approaches

Therapy	Mean (SD)						Infants, No.	Incremental Cost, $	Incremental Effectiveness	ICER	Peanut Allergy at Conclusion of Model, %
	Cost, $	Effectiveness, QALY	NMB, $	Food Allergic Reactions, No./Individual	Severe Allergic Reactions, No./Individual^a^	Deaths Due to Food Allergy, Rate/Patient					
No screening	13 449 (38 163)	29.25 (3.28)	2 912 020 (338 854)	1.07 (3.15)	0.53 (1.66)	0.00002 (0.0045)	100 000	NA	0.02	NA	6.3
SPT screening	15 279 (38 995)	29.23 (3.30)	2 908 022 (342 598)	1.01 (3.02)	0.52 (1.62)	0.00002 (0.0045)	100 000	$1830	NA	Dominated	6.5

Abbreviations: ICER, incremental cost-effectiveness ratio; NA, not applicable; NMB, net monetary benefit; QALY, quality-adjusted life-year; SPT, skin prick test.

^a^ When inclusive of only accidental anaphylaxis together with index reactions, including respiratory or cardiovascular compromise, the mean (SD) for no screening vs screening was 0.50 (1.59) vs 0.49 (1.47).

### Sensitivity Analyses

**Figure 2.**
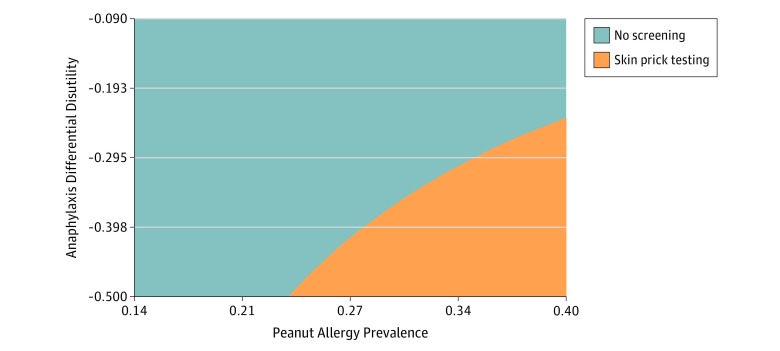
Deterministic Sensitivity Analyses Sensitivity analysis of the interaction between differential disutility of in-clinic or at-home index anaphylaxis and peanut allergy prevalence in high-risk infants at the threshold willingness to pay of $100 000/quality-adjusted life-year. Health disutility is a negative detriment of an allergic reaction (every −0.1 = 36.5 days of life in a year traded to avoid a reaction).

**Figure 3.**
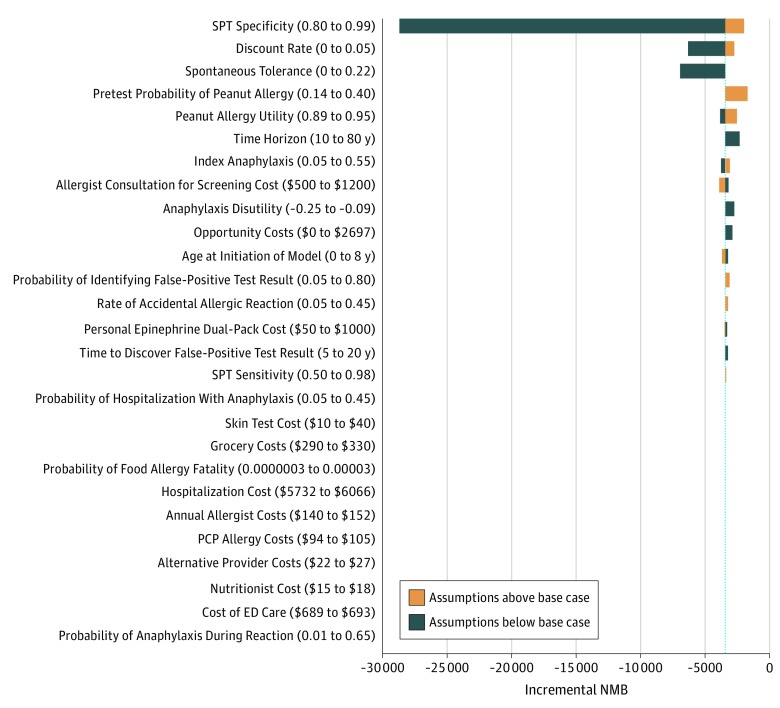
Tornado Diagram of Incremental Net Monetary Benefit (NMB) Deterministic sensitivity analyses across incremental NMB (a metric that synthesizes cost with monetization of the gain in quality-adjusted life-years [QALYs] so that higher NMB represents a higher-value intervention) for skin prick testing (SPT) vs no testing (willingness-to-pay threshold, $100 000/QALY). ED indicates emergency department; PCP, primary health care professional.

Because screening may incorporate variations in care that are sensitive to patient preference, additional analyses explored differential caregiver health utility and disutility. In deterministic sensitivity analyses at base-case sensitivity and specificity rates, SPT could be cost-effective (willingness to pay, $100 000/QALY) when applying a very high rate of disutility for a home reaction vs an in-clinic index reaction, in combination with a very high baseline peanut allergy prevalence in the high-risk infant with no peanut exposure (which exceeded the base-case utility difference for index in-clinic vs at-home anaphylaxis), and this interaction of these levers is demonstrated in Figure 2. We also considered the threshold for test sensitivity and specificity at which this analysis could be cost-effective. If an equivalent rate and disutility of accidental and index anaphylaxis was assumed with an 8-mm SPT sensitivity of 0.85 and specificity of 0.98, the screening approach became cost-effective at a peanut allergy prevalence of 36% (eFigure 1 in the Supplement). Additional deterministic analyses did not demonstrate cost-effective care for a screening approach (Figure 3). In probabilistic sensitivity analysis during a 20-year time horizon (n = 10 000), a no-screen approach was the optimal strategy in 99.9% at a willingness to pay of $100 000/QALY (eFigure 2 in the Supplement).

## Discussion

The previous analysis by Shaker et al^14^ comparing the US, UK, and Australia and New Zealand approaches calculated the cost to prevent a single case of a severe peanut allergic reaction under the pathway recommended by addendum 1 in the NIAID guidelines to be approximately US $101 963. Despite the lack of benefit, such a decision to screen may be preference sensitive. In a 2018 nationally representative survey of expecting parents (n = 1000) and new caregivers of infants (n = 1000),^15^ 61% had no or minimal concern for their child developing a food allergy, 54% thought early introduction mattered in terms of preventing food allergy development, 31% were willing to introduce peanut before 6 months of age, and 51% were unwilling to allow in-office risk assessments of peanut allergy (eg, allergy testing or oral challenge) before 11 months of age. Although this study was not designed as a formal preference-elicitation study and was performed at the beginning of the life cycle of the NIAID addendum guidelines, this population was representative of caregivers and family potentially having to make such a choice and infers that differential preference may exist regarding early peanut introduction.

This present analysis confirms previous observations that screening high-risk infants for peanut allergy is not cost-effective through a different model incorporating a longer time horizon and the potential for spontaneous discovery of false-positive test results. Given that change to this policy is unlikely, we took a novel approach of identifying key levers that could influence the cost-effectiveness of the existing NIAID policy, albeit with a narrow potential (and arguably an infeasible one given the specifications of the identified levers).^14^ These levers are a very high prevalence of preexisting peanut allergy in the infants undergoing early introduction, a high disutility for having an at-home index reaction, and enhanced SPT accuracy. Under most assumptions, even when differential in-clinic vs at-home anaphylaxis disutility is modeled, false-positive diagnoses are not subjected to ongoing risks of true peanut allergy, and spontaneous discovery of overdiagnosis is considered, the burden imposed by screening SPT simply overshadows a no-screening approach in terms of cost and effectiveness assessed by QALYs. In the present model, rates of anaphylaxis occurring with no screening being performed are marginally greater at a mean (SD) of 0.50 (1.59) vs 0.49 (1.47), and although this difference is not significant, this finding may reflect the current paradigm of advising that infants who are highly sensitized to peanut and have strong positive test results not be offered the opportunity to establish oral tolerance to prevent the risk that they may react at ingestion.^14,32^ However, this approach has not actually proven to be a shared decision consistent with caregiver (as opposed to health care professional) values. In actuality, it may be more accurate to assume that without being given any other option, such children with strong peanut sensitization are highly likely to develop peanut allergy if not offered peanut in the first year of life, as evidenced by the data (and secondary analysis data) from the LEAP study.^11^

A large contributor to the poor cost-effectiveness of the screening approach relates to the poor accuracy of peanut allergy diagnostic test results in children who have not directly ingested peanut and developed symptoms of an allergic reaction.^33,34^ For this reason, multiple past food allergy guidelines in the United States and elsewhere have urged caution in testing individuals who have not ever eaten a food before, a situation representing exceptionally low pretest probability at worst and marginal pretest probability (which was elevated to marginal through severe eczema and/or egg allergy as factors that may increase the odds of someone having peanut allergy compared with the general population) at best.^1,35^

An optimal screening test should maximize the ability to accurately identify disease that would lead to harm if not otherwise diagnosed early (eg, test sensitivity), because of adverse consequences due to the natural history of the disease or because early management resulting from identification can prevent likely complications of the disease, while minimizing potential false-positive diagnoses and the resulting harm from managing a condition that is not actually present (eg, test specificity). However, it is not clear that such situations exist in the realm of food allergy screening before introduction, given that the test precision is imperfect (the test is most interpretable when results are negative and is difficult to interpret when results are positive, particularly at a 3-mm cutoff) and carries significant risk of false-positive results. Even when a patient is accurately diagnosed with peanut allergy early, no treatment (and certainly no cure) is available yet (nor will be available until the child is 4 years of age).^2^ Thus, early diagnosis may prevent an index reaction (including a potentially severe one) under certain circumstances as the only tangible benefit and may identify someone for future therapy, but otherwise, early diagnosis increases one’s life span with peanut allergy and adds time under which poor quality of life may develop as a realistic detriment. The advent of an available peanut allergy therapy in younger children might increase the value of peanut allergy screening.

To our knowledge, no published or anecdotal reports exist of deaths due to peanut anaphylaxis during infancy related to early introduction of a small amount of peanut protein (even in an infant considered at high risk for peanut introduction). With an increase in early introduction, ongoing surveillance of fatal food reactions during infancy will be important to continue to evaluate the safety of this practice. In the HealthNuts study, although anaphylaxis rarely occurred, most reactions were mild and cutaneous.^12^ A similar finding was noted in LEAP, although the trial was right censored from challenge-proven outcomes at an SPT result of 5 mm, as well as in the other early introduction studies.^6^ Most importantly, no data are available to suggest that death due to food allergy is more likely in this age than any other age.^36^ One potential approach is to presume that this is a preference-sensitive choice, develop a decision aid, and provide families with a clear understanding of risks and benefits of each approach that allows them to make the best decision for themselves. Although this will not change the fact that, when viewed through an economic lens, peanut allergy screening for early introduction is not cost-effective, it may help offset some potentially low-value care if parents can be given more options than the current NIAID guidelines suggest.

### Limitations

This analysis is limited given that it is a simulation reliant on the quality of its inputs, which in this case come from the LEAP and HealthNuts cohorts. Because those studies were not conducted in a US population, there could be difficulty with generalizability; nonetheless, they represent the most robust and reasonable inputs for this model.^6,12,13^ In addition, the precise health utilities for peanut allergy, early introduction, or preference for home vs supervised initial reactions are unknown and need to be established. We explored levers that we believed might be amenable to change, but there could be other levers that we did not explore. We did not include risks of motor vehicle collision fatality associated with transportation for allergy evaluations, which could in fact greatly overshadow any screening-associated risk reduction for death due to anaphylaxis.^36,37,38^ Also, as was noted in the previous analysis by Shaker et al,^14^ we did not model wait lists for health care professionals and reduced access as factors that could affect decision-making and the ability to receive timely care.

## Conclusions

In this study, screening for peanut sensitization in high-risk infants and presumptively diagnosing the child with a peanut allergy based on large SPT result size or only providing the option for in-clinic introduction for those with small- to moderate-size SPT results was not found to be cost-effective compared with the general permissive strategy of recommending early introduction at home without any assessment. However, it appears that this strategy could be cost-effective if caregivers have a strong health utility for having an index reaction occur under medical supervision, with a very accurate test result, or at a very high rate of ambient preexisting peanut allergy before early introduction occurs. Further research is needed to better define these key attributes, and presuming differential health utility exists for where caregivers prefer index reactions to occur, a formal decision aid could be of considerable use to help caregivers and health care professionals engage in shared decision-making to facilitate early peanut introduction.

## Notes

Supplement. **eTable.** NIAID Addendum Guidelines for Early Peanut Introduction **eFigure 1.** Deterministic Sensitivity Analyses **eFigure 2. **Cost-effectiveness Acceptability
