# Supplementary material for: Determining Levers of Cost-effectiveness for Screening Infants at High Risk for Peanut Sensitization Before Early Peanut Introduction
Source: JAMA Netw Open. 2019 Dec 20;2(12):e1918041. doi: 10.1001/jamanetworkopen.2019.18041 (PMC6991237; doi:10.1001/jamanetworkopen.2019.18041)
Supplement: Supplement. — eTable. NIAID Addendum Guidelines for Early Peanut Introduction eFigure 1. Deterministic Sensitivity Analyses eFigure 2. Cost-effectiveness Acceptability [file jamanetwopen-2-e1918041-s001.pdf]

Supplementary Online Content

Greenhawt M, Shaker M. Determining levers of cost-effectiveness for screening infants at high risk for peanut sensitization before early peanut introduction. *JAMA Netw Open.* 2019;2(12):e1918041. doi:10.1001/jamanetworkopen.2019.18041

eTable. NIAID Addendum Guidelines for Early Peanut Introduction
eFigure 1. Deterministic Sensitivity Analyses
eFigure 2. Cost-effectiveness Acceptability

This supplementary material has been provided by the authors to give readers additional information about their work.

© 2019 Greenhawt M et al. *JAMA Network Open.*

eTable. NIAID Addendum Guidelines for Early Peanut Introduction

**Addendum Guideline**	**Infant Criteria**	**Recommendations**	**Earliest Recommended Age of Peanut Introduction**
1	Severe eczema, egg allergy or both	Strongly consider evaluation by sIgE and/or SPT, and if necessary an oral food challenge. Based on test results, introduce peanut containing foods	4 to 6 months
2	Mild to moderate eczema	Introduce peanut-containing foods	Around 6 months
3	No eczema or any food allergy	Introduce peanut-containing foods	Timing of introduction is age appropriate and in accordance with family preferences and cultural practices

© 2019 Greenhawt M et al. *JAMA Network Open.*

eFigure 1. Deterministic Sensitivity Analyses

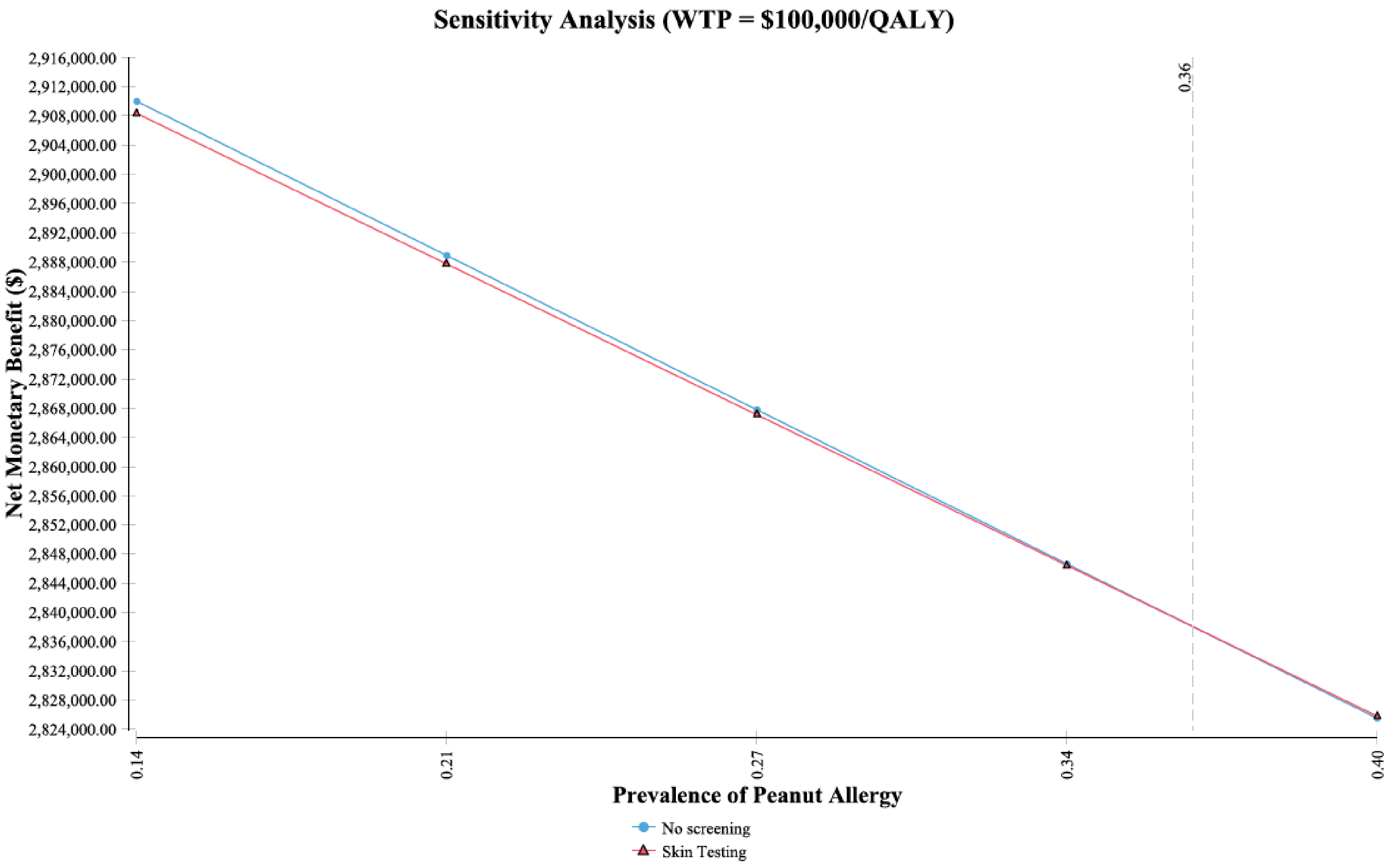

One-way sensitivity analysis of peanut allergy prevalence at equivalent rates and disutilities of index and accidental anaphylaxis rates with SPT sensitivity of 0.85 and specificity of 0.99.

© 2019 Greenhawt M et al. *JAMA Network Open.*

eFigure 2. Cost-effectiveness Acceptability

Figure e1a

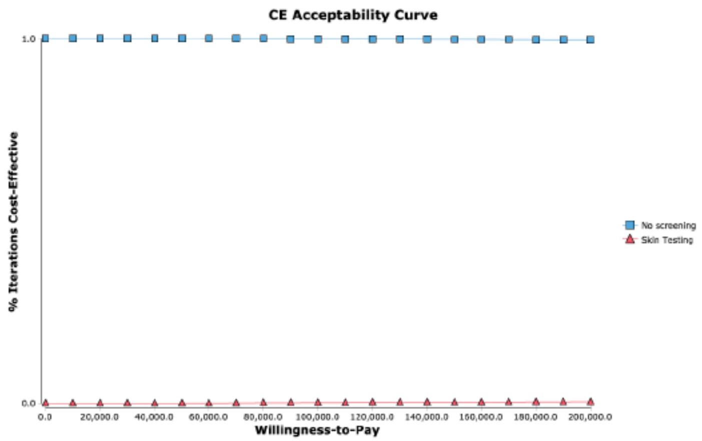

Figure e1b

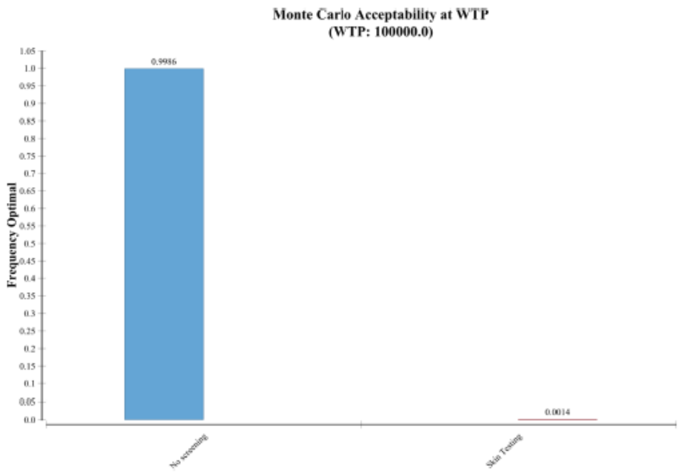

In probabilistic sensitivity analyses over a 20 year horizon (n=10,000) a skin test screening strategy was not cost-effective in 99.9% of simulations (WTP = $100,000/QALY)

© 2019 Greenhawt M et al. *JAMA Network Open.*
